# Atomistic Insights into the Molecular Interactions of Rod and Cluster Shaped CdS for Photocatalytic Water Splitting

**DOI:** 10.3390/molecules31010092

**Published:** 2025-12-25

**Authors:** Aliya Assilbekova, Irina Irgibaeva, Mirat Karibayev, Ayaulym Amankeldiyeva, Sergei Piskunov, Nurlan Almas, Galiya Baisalova, Anuar Aldongarov

**Affiliations:** 1 Department of Technical Physics, L.N. Gumilyov Eurasian National University, Astana 010000, Kazakhstan; asylaliya@yandex.kz (A.A.); irgsm@mail.ru (I.I.); nurlanalmasov@gmail.com (N.A.); 2 Laboratory of Renewable Energy, National Laboratory Astana, Nazarbayev University, Astana 010000, Kazakhstan; mirat.karibayev@nu.edu.kz (M.K.); ayaulym.amankeldiyeva@nu.edu.kz (A.A.); 3 Institute of Solid State Physics, University of Latvia, 1000 Riga, Latvia; piskunov@lu.lv; 4 Department of Chemistry, L.N. Gumilyov Eurasian National University, Astana 010000, Kazakhstan

**Keywords:** cadmium sulfide, rod and cluster morphology, photocatalytic water splitting, density functional theory, molecular dynamics

## Abstract

Understanding the atomic-level behavior of photocatalysts under hydrated conditions is essential for improving hydrogen production efficiency. In this work, density functional theory calculations and classical all-atom molecular dynamics simulations were performed to investigate the intra- and intermolecular interactions of rod- and cluster-shaped cadmium sulfide in the presence of implicit and explicit water, respectively. The density functional theory optimized geometries, reduced density gradient, noncovalent interaction, critical point, and molecular electrostatic potential maps were examined using the LC-ωPBE functional with the LANL2DZ basis set and the IEFPCM implicit solvation model, while explicit hydration was modeled via classical all-atom molecular dynamics simulations by obtaining molecular snapshots and radial distribution functions. Density functional theory results revealed that rod-shaped cadmium sulfide exhibits stronger directional bonding and higher electronic localization compared to cluster-shaped cadmium sulfide, while classical all-atom molecular dynamics simulations showed that water molecules preferentially interact with surface S atoms of cadmium sulfide sites. This atomistic insight clarifies how morphology and hydration jointly modulate cadmium sulfide electronic structure and reactivity, providing guidance for the rational design of efficient cadmium sulfide-based photocatalysts for solar-driven water splitting.

## 1. Introduction

The increasing global demand for sustainable and carbon-neutral energy has intensified research on photocatalytic water splitting as a promising pathway for hydrogen production. Semiconductor-based photocatalysts have received particular attention due to their capability to directly convert solar energy into chemical fuels. Among various semiconductors, cadmium sulfide (CdS) stands out because of its narrow band gap (~2.4 eV), visible-light responsiveness, and strong absorption in the solar spectrum. CdS has been extensively studied in diverse morphologies such as nanoparticles, nanorods, nanowires, and quantum dots, each exhibiting distinct electronic and catalytic characteristics [1,2,3]. However, the underlying atomistic mechanisms that govern the interaction of CdS with surrounding molecules, especially water, remain insufficiently understood. Such molecular-level insights are crucial for improving photocatalytic performance and stability in water-splitting environments.

The photocatalytic activity of CdS is inherently linked to its morphology and electronic structure. Rod-shaped CdS nanostructures often exhibit efficient charge separation along their longitudinal axis, enabling enhanced migration of photogenerated carriers and reduced recombination losses. In contrast, cluster-shaped CdS nanoparticles possess a higher density of surface atoms, which can act as active centers for adsorption and reaction processes but may also suffer from rapid electron-hole recombination or surface oxidation [4,5,6,7]. Understanding how these structural differences influence electron localization, charge transfer, and molecular interactions is essential for rational catalyst design. Moreover, water molecules not only serve as reactants in the photocatalytic process but also dynamically interact with CdS surfaces, altering local charge distributions, hydrogen-bond networks, and interfacial stability [8,9,10,11]. To summarize the key structural and functional distinctions relevant to this study, a comparative overview of both morphologies and their interaction behavior with water is presented in Table 1.

The information summarized in Table 1 highlights that both shapes contribute distinct advantages to photocatalytic hydrogen production. Understanding these morphological and interfacial effects at the atomistic level is therefore essential to rationalize the differences in electronic structure, charge transfer, and stability explored in this work. Despite extensive experimental investigations, probing such interactions at the atomic scale remains challenging due to the transient and complex nature of solvent-semiconductor interfaces [19,20,21,22,23,24,25]. In this context, density functional theory (DFT) and classical all-atom molecular dynamics (MD) simulations provide a powerful computational framework to examine electronic structure and molecular interaction of photocatalytic systems.

In the present work, we perform detailed DFT calculations and classical all-atom MD simulations to elucidate the molecular interactions of rod- and cluster-shaped CdS in the presence of implicit and explicit water molecules. The following sections present a detailed analysis of the DFT results for the implicit-water case including optimized structures, reduced density gradients (RDG), noncovalent interactions (NCI), critical points, and molecular electrostatic potential (MEP) maps, followed by the molecular snapshots and radial distribution functions (RDF) from classical all-atom MD simulations for the explicitly solvated water case, the methodology, and finally the conclusions.

## 2. Results and Discussion

### 2.1. DFT Calculation Results and Discussion

#### 2.1.1. Optimized Structures

**Figure 1:**
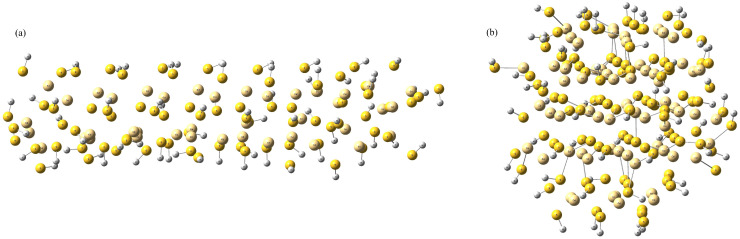
Optimized structure of (**a**) rod-shaped CdS, (**b**) cluster-shaped CdS, both optimized with implicit water. Color scheme: light yellow (cadmium); dark yellow (sulfur); and white (hydrogen).

Figure 1 illustrates the optimized geometries of the rod-shaped and cluster-shaped CdS structures obtained under implicit water solvation. Both morphologies preserve the characteristic Cd–S bonding network of wurtzite-type CdS; however, their local coordination environments and surface relaxation behaviors differ noticeably. The rod-shaped system exhibits a more ordered and extended CdS framework along the crystallographic growth axis, leading to relatively uniform Cd–S bond distances and S–Cd–S angles. In contrast, the cluster-shaped CdS undergoes more pronounced geometric relaxation after optimization, reflecting its higher proportion of under-coordinated surface atoms. This results in slightly shorter Cd–S bonds and broader angular distributions at the surface.

Implicit hydration stabilizes both morphologies by screening surface charge irregularities, but its effect is more significant in the cluster system, where solvation reduces geometric distortions associated with dangling bonds. The differences in bond lengths and angles between rod and cluster geometries directly influence their electronic structure, potentially affecting charge separation efficiency and surface-active sites during photocatalytic water splitting (Table 2, see Supplementary Materials).

These structural observations provide crucial atomistic insight into how morphology and hydration modulate CdS reactivity, thereby informing the rational design of shape-dependent photocatalysts.

#### 2.1.2. Reduced Density Gradient and Noncovalent Interactions

**Figure 2:**
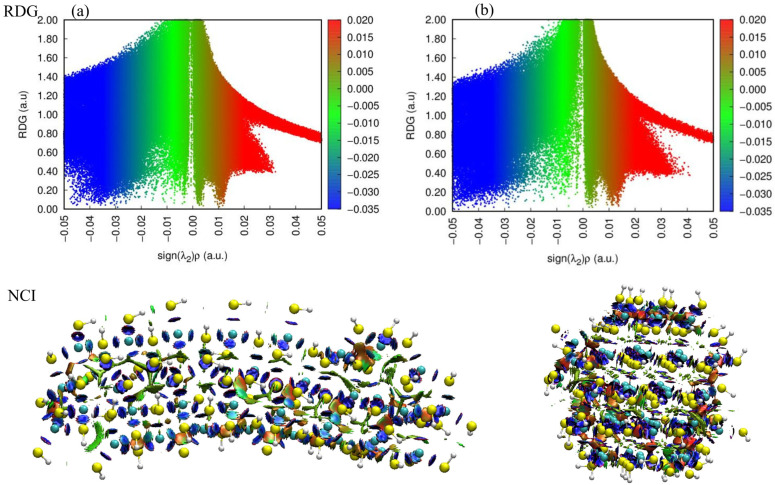
RDGs and NCIs of (**a**) rod-shaped CdS with implicit water, (**b**) cluster-shaped CdS, both optimized with implicit water. Color scheme of atoms: cyan (cadmium), yellow (sulfur), and white (hydrogen). Color scheme of RDG and NCI: blue (strong attraction), green (van der Waals interaction), and red (strong repulsion).

Figure 2 illustrates the nature and strength of NCI for rod-shaped (a) and cluster-shaped (b) CdS systems optimized with implicit water. The analysis utilizes the sign of the second Hessian eigenvalue multiplied by the electron density, plotted against the RDG. This method effectively separates interactions into three distinct regimes, as detailed in the color scale legend: strong attraction (blue), Van der Waals interactions (green), and strong steric repulsion (red). In the scatter plots for both the rod and cluster configurations, the vertical spikes represent areas of low-density gradient, which correspond to the presence of interactions.

In Figure 2a (rod-shaped), a significant region of strong attraction is visible on the negative side of the x-axis. Specifically, the dense blue scattering appears in the region where the sign of the second Hessian eigenvalue ranges from approximately −0.05 to −0.02 a.u. These spikes indicate strong attractive forces, likely attributable to electrostatic interactions within the ionic CdS lattice and hydrogen bonding interactions with the implicit solvent environment. Simultaneously, a broad green region is observed centered near 0.00 a.u., extending roughly between −0.01 and +0.01 a.u. This corresponds to the green isosurfaces visible in the 3D molecular representation below the plot. These extensive green surfaces suggest that Van der Waals forces play a critical role in stabilizing the elongated rod structure. The presence of red spikes on the positive side of the *x*-axis, extending from +0.01 to +0.05 a.u., signifies steric repulsion. In the rod-shaped 3D model, these are visualized as red patches, often found within the center of ring structures where electron density is crowded (the “steric effect in ring and cage”). Figure 2b (cluster-shaped) exhibits a similar tripartite distribution but with notable morphological differences in the 3D NCI plot. The scatter plot maintains the strong attractive spikes (blue) near −0.04 a.u. and the Van der Waals region (green) near zero. However, the globular nature of the cluster results in a more condensed arrangement of steric repulsion zones. The 3D visualization for the cluster shows a complex network of red isosurfaces deeply embedded within the core, consistent with the higher steric hindrance expected in a crowded, cage-like geometry.

The coexistence of strong attractive peaks (blue) and Van der Waals interactions (green) in the RDG plots for both systems confirms their structural stability in the implicit water medium, balanced against the inevitable steric repulsions (red) inherent to the atomic packing.

#### 2.1.3. Critical Points

**Figure 3:**
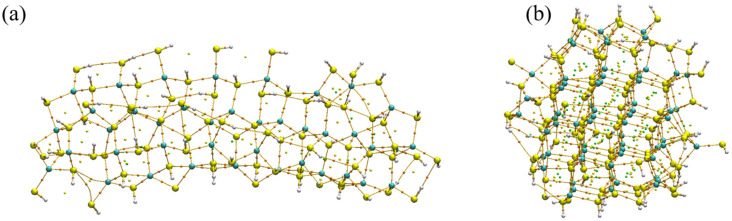
Critical points of (**a**) rod-shaped CdS with implicit water, (**b**) cluster-shaped CdS, both optimized with implicit water. Color scheme: orange (bond critical points); cyan (cadmium); yellow (sulfur); and white (hydrogen).

Figure 3 shows the bond critical points (BCPs) identified for the rod- and cluster-shaped CdS models optimized with implicit water. The BCPs are concentrated along Cd–S linkages in the interior of the rod, whereas the cluster exhibits a larger spread of BCPs with a higher density near under-coordinated surface sites. Electron density at Cd–S BCPs (ρ) is higher in the rod model than in the cluster, reflecting the more uniform, bulk-like Cd–S bonding in the rod and the greater surface relaxation in the cluster (Table 3).

The Laplacian values (∇^2^ρ) at the Cd–S BCPs are negative or only slightly positive, indicating accumulation of electron density between the Cd and S centers and a partially shared (covalent/closed-shell mixed) character. Ellipticity (ε) at these BCPs is small, consistent with largely cylindrically symmetric Cd–S bonds and limited π-type distortion. Using the potential energy density V(r) at the BCP and Espinosa’s approximation, estimated interaction energies for representative Cd–S contacts are significantly larger (more stabilizing) in the rod than in the cluster model, indicating stronger localized bonding in the rod interior. Together, these CP indicators confirm that rod-shaped CdS preserves bulk-like bonding motifs, while the cluster shape shows weakened and more heterogeneous surface bonds, insights that correlate with the RDG/NCI maps in Figure 2 and that have implications for surface reactivity in photocatalysis.

#### 2.1.4. Molecular Electrostatic Potential Maps

**Figure 4:**
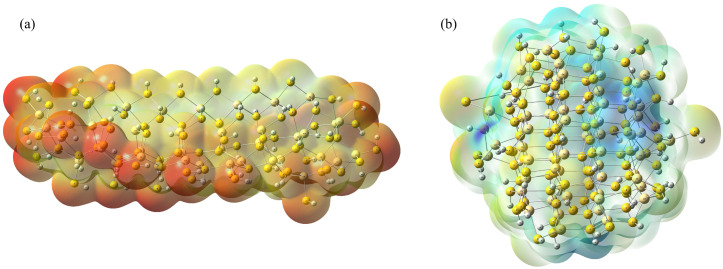
MEP maps of (**a**) rod-shaped CdS (magnitude from −0.23 a.u. to 0.23 a.u.), (**b**) cluster-shaped CdS (magnitude from −0.10 a.u. to 0.10 a.u.), both optimized with implicit water. Color scheme: light yellow (cadmium); dark yellow (sulfur); and white (hydrogen).

MEP analysis was performed on the CdS nanostructures optimized in implicit water. The MEP maps, presented in Figure 4, visualize the charge distribution across the molecular surfaces, allowing the identification of electron-rich (nucleophilic) and electron-deficient (electrophilic) sites. The color code ranges from red (negative potential) to blue (positive potential).

Figure 4a displays the MEP of the rod-shaped CdS. The surface is predominantly covered by red and orange regions, indicating a strong negative electrostatic potential with a magnitude reaching −0.23 a.u. This extensive negative surface charge implies a high electron density, likely localized around the surface sulfur atoms.

In contrast, the cluster-shaped CdS in Figure 4b reveals a distinct charge separation, directly addressing the question regarding polarization. The map shows a heterogeneous distribution, with significant blue patches (positive potential) intermixed with green and yellow areas (neutral to slightly negative), within a narrower range of ±0.10 a.u. This variation confirms that considerable polarization occurs within the cluster geometry. The implicit solvent calculation highlights that, while the rod maintains a strong overall negative surface potential, the cluster exhibits a polarized surface with distinct positive and negative poles, which is critical for understanding its specific electrostatic interactions in an aqueous environment.

Recent experimental and theoretical studies further highlight the importance of interfacial charge-management strategies in CdS-based photocatalysts. For example, Wu et al. demonstrated that constructing S-scheme CdS QD/In_2_O_3_ heterojunctions significantly suppresses carrier recombination and enhances H_2_ evolution by over an order of magnitude, underscoring the critical role of controlled charge separation pathways [26]. Similarly, Zhang et al. reported that Mn_0.3_Cd_0.7_S/CoPB Schottky junctions featuring interfacial Co–S bonding greatly promote directional electron transfer and photothermal-assisted activity, emphasizing how engineered interfaces can stabilize charge flow, an effect consistent with our observation that rod-shaped CdS favors more efficient charge separation than cluster morphologies [27].

### 2.2. Classical All-Atom MD Simulation Results and Discussion

#### 2.2.1. Classical All-Atom MD Simulation Snapshots

**Figure 5:**
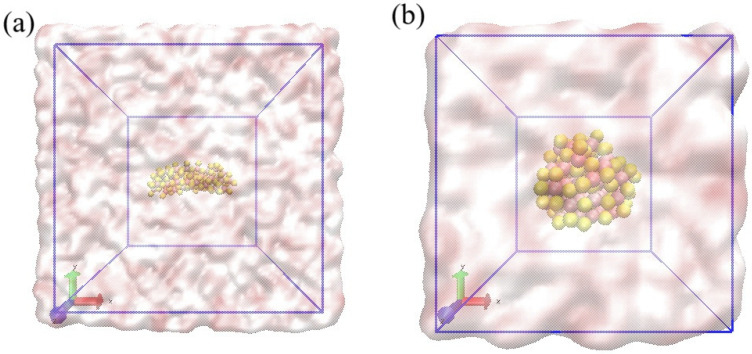
Molecular Dynamics simulation snapshots of (**a**) rod-shaped CdS, (**b**) cluster-shaped CdS. Color scheme: reddish (cadmium); white (hydrogen); and yellow (oxygen), white red transparent isosurface (explicitly solvated water molecules).

Figure 5 illustrates the simulation systems used for the classical all-atom MD study. Unlike the implicit solvent models utilized in the quantum mechanical calculations, these snapshots depict the rod-shaped (a) and cluster-shaped (b) CdS nanostructures fully immersed in an explicit aqueous environment.

The nanoparticles are centered within cubic simulation boxes under periodic boundary conditions, ensuring a continuous bulk solvent phase. The transparent red-and-white isosurfaces represent the explicitly solvated water molecules, which completely encapsulate the CdS surfaces. This setup is crucial for capturing the realistic dynamic behavior of the system, allowing for the subsequent analysis of RDFs.

#### 2.2.2. Radial Distribution Functions

**Figure 6:**
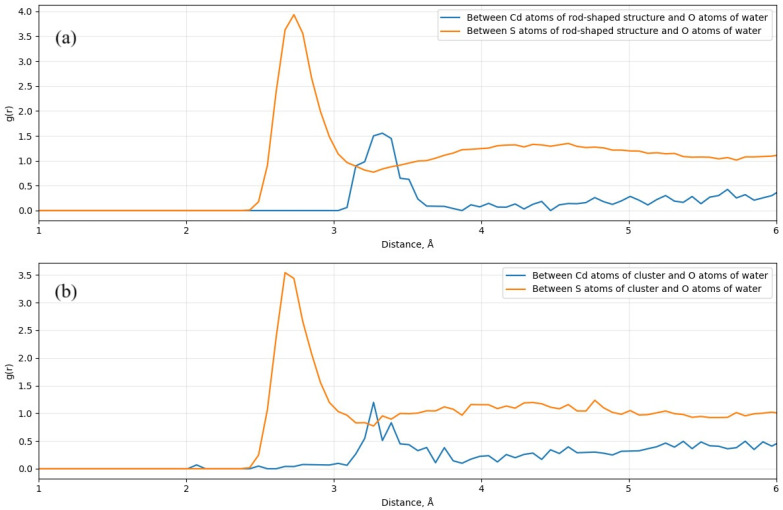
RDFs of (**a**) rod-shaped CdS, (**b**) cluster-shaped CdS.

The local structure of the solvent surrounding the nanoparticles was investigated using Radial Distribution Functions (RDFs), g(r), as shown in Figure 6. These RDFs quantify the probability of finding water oxygen atoms of water at specific distances from the Cadmium (Cd) and Sulfur (S) atoms of the nanostructures. The most prominent feature in both plots is the interaction between the surface sulfur atoms and water oxygen, indicated by the orange lines. For the rod-shaped CdS nanostructure (Figure 6a), the S–O interaction exhibits a sharp, intense peak centered at a radial distance of approximately 2.7 Å. This short bond distance reflects strong hydrogen bonding or electrostatic interactions between water molecules and surface sulfur atoms. In contrast, the Cd–O interaction (blue line) appears as a broader and less intense peak at a larger distance of around 3.3 Å, suggesting that water molecules are less tightly bound to cadmium sites or predominantly reside in a secondary solvation shell.

A comparison of the different morphologies indicates that the rod-shaped structure demonstrates stronger solvation behavior than the cluster-shaped counterpart. Peak intensity in the RDFs serves as a quantitative measure of interaction strength and local ordering. In the rod-shaped system, the S–O peak reaches a maximum value of approximately 3.9, whereas the cluster-shaped system (Figure 6b) exhibits a lower peak intensity of 3.5. Similarly, the Cd–O interaction is well-defined in the rod (g(r)≈1.5) but significantly weaker and more irregular in the cluster (g(r)≈1.2). The reduction in peak heights in the cluster indicates a more disordered solvent structure and weaker hydrophilic interactions (see Supplementary Materials). These observations confirm that the rod-shaped CdS geometry promotes stronger, more ordered hydration, highlighting the critical role of nanoparticle morphology in determining solvent structuring at the molecular level.

### 2.3. Comparison with Experimental Work

The computational results obtained in this work show clear morphology-dependent behavior that aligns with experimental observations on CdS nanostructures. Rod-shaped CdS exhibits more uniform charge distribution along its axis and maintains structural stability when interacting with water, which corresponds well with reports that rod-like CdS materials display higher photocatalytic activity and improved optical performance in aqueous systems. In contrast, cluster-shaped CdS shows stronger local interactions with surrounding molecules and more heterogeneous charge localization, matching experimental findings that CdS nanoparticles, although rich in active surface sites, often experience faster recombination and reduced long-term stability.

These correlations (Table 4) are consistent with the literature, indicating that CdS nanorods generally outperform nanoparticles in hydrogen evolution experiments, doped and structured CdS exhibit improved visible-light response, and morphology plays a crucial role in photocatalytic durability and efficiency [28,29,30,31].

## 3. Materials and Methods

### 3.1. Theoretical Model and Designed System

**Figure 7:**
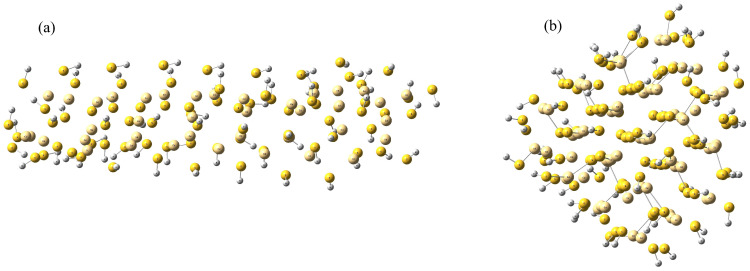
Three-dimensional structures of (**a**) rod-shaped, (**b**) cluster-shaped CdS. Color scheme: light yellow (cadmium); dark yellow (sulfur); and white (hydrogen).

To investigate the molecular-level behavior of cadmium sulfide (CdS) with different morphologies, two representative models—rod-shaped and cluster-shaped CdS—were constructed (Figure 7). These geometries were designed to mimic experimentally observed CdS nanostructures that exhibit distinctive photocatalytic activities depending on their morphology. The rod-shaped CdS represents an elongated configuration with an extended Cd–S network, while the cluster-shaped CdS corresponds to a more compact assembly of Cd and S atoms with higher surface curvature.

Both CdS systems were optimized in the presence of implicit water. The rod-shaped and cluster-shaped CdS models were constructed by truncating the hexagonal wurtzite CdS crystal structure, the thermodynamically stable polymorph under photocatalytic conditions. The rod comprises 40 Cd and 80 S atoms (with hydrogen passivation of dangling bonds), while the cluster contains 65 Cd and 99 S atoms, as listed in the Supporting Information. The final model sizes represent the smallest clusters that retain realistic Cd–S bond lengths and S–Cd–S angles. All optimized structures were confirmed as true minima with no imaginary frequencies, and no reconstruction or phase change was observed. Hydrogen passivation and symmetric truncation were applied to minimize edge effects inherent to finite non-periodic models.

### 3.2. DFT Calculation Methodology

The LC-ωPBE functional was employed due to its reliable treatment of long-range exchange interactions and accurate description of semiconductor band structures. The LANL2DZ basis set [32,33] was used for all atoms, as it effectively incorporates relativistic effective core potentials (ECPs) suitable for heavy elements such as cadmium.

To simulate solvation effects, an implicit solvent model was included using the Self-Consistent Reaction Field (SCRF) approach with the Integral Equation Formalism Polarizable Continuum Model (IEFPCM), where water was defined as the solvent in all cases. This setup provides a more realistic depiction of aqueous photocatalytic environments while maintaining computational efficiency. The combination of implicit solvation (PCM model) and explicit single water molecule allows for simultaneous evaluation of bulk and localized hydration effects.

Geometry optimizations were conducted without imposing any symmetry constraints using the opt keyword. Tight SCF convergence criteria (SCF = XQC) were applied to ensure energy convergence and stability of the electronic wavefunction. All optimized structures were confirmed as true minima by verifying the absence of imaginary vibrational frequencies.

The atomic coordinates were initially built and visualized using GaussView 6, ensuring realistic configurations with minimized steric hindrance and proper Cd–S bonding geometries. These optimized structures were later used for in-depth analysis of reduced density gradient, noncovalent interactions, critical points, and electron localization function. Density Functional Theory (DFT) calculations were performed using the Gaussian 16 software package [34,35].

Post-optimization analyses were performed using Multiwfn 3.7 software [36,37], which enabled quantitative and visual examination of reduced density gradient (RDG) plots, non-covalent interaction (NCI) regions, topological features within the Quantum Theory of Atoms in Molecules (QTAIM) framework, and electron localization function.

This computational approach—combining explicit and implicit solvation, advanced long-range corrected DFT functionals, and topological electron density analyses—provides a robust foundation for understanding the structure–property relationship and photocatalytic efficiency of CdS with different morphologies.

### 3.3. Classical All-Atom MD Simulations Methodology

Classical all-atom MD simulations were carried out using the LAMMPS version: 7 February 2024 (Update 1) package, and VMD 1.9.1 software [38,39] to investigate two different CdS–H nanostructures immersed in explicit water: a spherical CdS–H nanocluster and a rod-shaped CdS–H structure. For both systems periodic boundary conditions were applied in all three directions.

A hybrid/overlay interaction scheme was used. The Cd–S framework in both the nanocluster and rod structure was described using the modified Stillinger–Weber (SW) potential, which incorporates the required three-body terms for Cd–Cd–S and S–Cd–S interactions [40,41]. Water molecules were modeled using the TIP3P potential [42], where harmonic O–H bonds and a fixed H–O–H angle of 104.52° were applied to maintain molecular geometry. Hydrogen atoms attached to surface sulfur atoms (H passivation) were treated via harmonic H–S bonds and Cd–S–H angles. Nonbonded interactions between water and the inorganic components were represented using Lennard-Jones with Coulombic interactions with TIP3P-consistent parameters assigned to oxygen and with O–S cross-interactions defined through LJ mixing rules. The H–S and Cd–water LJ terms were set to zero to avoid nonphysical attractions. Long-range electrostatic forces were computed using the PPPM method.

To keep the inorganic structures rigid, all Cd and S atoms were grouped and immobilized with zero initial velocities. This ensured that both the spherical and rod-shaped CdS–H structures remained fixed, allowing only the surrounding TIP3P water to evolve dynamically.

Two CdS–H nanostructures (rod and cluster) were constructed and solvated in explicit water. Cadmium (Cd) atoms are shown in pink, sulfur (S) atoms in yellow. Water molecules were rendered transparent to highlight the solute geometry. Both systems were equilibrated prior to production runs, with simulation box sizes chosen to accommodate their distinct morphologies: the compact cluster was placed in a cubic box of 40 Å per side, while the elongated rod-shaped nanostructure required a larger cubic box of 80 Å per side to avoid self-interaction across periodic boundaries.

Each system underwent energy minimization using a conjugate-gradient algorithm before equilibration. Production simulations were performed in the NVT ensemble at 298 K using a Nosé–Hoover thermostat with a 100 fs relaxation time. A timestep of 1 fs was used, and neighbor lists were updated every timestep with a 2.0 Å skin distance. Thermodynamic quantities were recorded every 100 steps, and the total production time for each system was 10 ns.

## 4. Conclusions

Based on the comprehensive atomistic investigation conducted in this study, we conclude that the morphology of CdS nanostructures plays a decisive role in modulating their structural stability, electronic properties, and interfacial interactions with water, which collectively influence photocatalytic performance for water splitting.

Density functional theory (DFT) calculations revealed that rod-shaped CdS maintains a more ordered and anisotropic bonding network with stronger directional Cd–S interactions and higher electron localization, favoring efficient charge separation along its longitudinal axis. In contrast, cluster-shaped CdS exhibits greater surface relaxation, heterogeneous charge distribution, and weakened surface bonds, which may promote charge recombination. Molecular electrostatic potential (MEP) maps further confirmed that rod-shaped CdS possesses a uniformly negative surface, while cluster-shaped CdS shows localized charge polarization.

Classical all-atom molecular dynamics (MD) simulations of explicitly solvated systems demonstrated that water molecules interact more strongly and orderly with rod-shaped CdS, particularly through S–O hydrogen bonding, as evidenced by sharper and more intense radial distribution function (RDF) peaks. The cluster-shaped system exhibited less structured hydration and weaker solvent interactions, correlating with its more disordered surface.

These computational insights align with experimental trends where rod-like CdS morphologies generally exhibit superior photocatalytic activity and stability compared to nanoparticle clusters. The integrated DFT and MD approach clarifies that the synergistic combination of morphological integrity, controlled charge separation, and ordered hydration shells makes rod-shaped CdS a more promising candidate for efficient and durable hydrogen evolution.

However, these findings are limited by the reliance on static DFT calculations and simplified CdS photocatalysts in implicit water models, which cannot fully capture explicit solvent effects. In this work, we also employed classical all-atom MD simulations to study intermolecular interactions between explicitly solvated water and CdS; however, this approach cannot model the creation and breaking of chemical bonds on the CdS surface in the presence of explicit water molecules. Additionally, because finite CdS clusters were used rather than periodic surfaces, some degree of edge-induced artifacts may remain despite geometric optimization and hydrogen passivation. Reactive all-atom MD simulations using ReaxFF or ab initio molecular dynamics (AIMD) were not implemented in the present study. Future work should incorporate AIMD or reactive force field-based simulations to capture time-dependent hydration behavior, bond rearrangements, and more complex photocatalytic processes.

## Figures and Tables

**Table 1:** Comparative overview of rod- and cluster-shaped CdS morphologies [11,12,13,14,15,16,17,18].

Aspect	Rod-Shaped CdS	Cluster-Shaped CdS	Effect of Water & Intermolecular Interactions	Photocatalytic Implications
Morphology	Elongated 1D structures with anisotropic charge transport.	Nearly spherical or aggregated nanoparticles with higher surface area.	Water molecules form hydrogen bonds, influencing aggregation and surface stability.	Rods favor charge separation; clusters offer abundant active sites.
Synthesis	Typically obtained via hydro/solvothermal routes with controlled precursor ratios.	Formed under similar conditions with variations in solvent or capping agents.	Solvent polarity and hydration modulate nucleation and surface chemistry.	Synthesis control enhances morphology and stability for catalysis.
Electronic/Optical	Band gap ≈ 2.4 eV; efficient visible-light absorption; reduced recombination.	Comparable gap but higher carrier recombination due to boundaries.	Water alters surface charge density, affecting carrier dynamics.	Charge separation efficiency governs hydrogen evolution rate.
Stability	Enhanced by surface passivation and uniform growth.	More prone to photocorrosion from surface defects.	Hydration can either stabilize or promote surface oxidation.	Stability improved via heterojunctions or protective coatings.
Hydrogen Evolution	Active rod tips enhance catalytic sites and electron transfer.	High surface area aids adsorption but needs recombination control.	Water acts as both reactant and stabilizing medium.	Regarding morphology, environment synergy dictates overall efficiency.

**Table 2:** Key structural parameters of optimized CdS models (implicit water).

Parameter	Rod-Shaped CdS	Cluster-Shaped CdS
Average Cd–S distance (Å)	2.53	2.49
Average S–Cd–S angle (°)	107.8	104.6
Range of Cd–S distances (Å)	2.50–2.57	2.42–2.54
Range of S–Cd–S angles (°)	103–112	95–110
Notable structural feature	Highly ordered anisotropic CdS chain	Higher surface relaxation and distortion

**Table 3:** Representative Cd–S BCP properties (Interaction energies estimated as $E\approx \frac{1}{2}V\left(r\right)\times 627.509$ kcal·mol^−1^).

System (Representative BCP)	Rod, Selected BCP	Cluster, Selected BCP
Bond length (Å)	2.53	2.49
ρ (a.u.)	0.16	0.05
∇^2^ρ (a.u.)	−0.27	+0.16
Ellipticity ε	0.0362	0.0002
V(r) (hartree)	−0.1795639	−0.0490795
Estimated E (kcal·mol^−1^)	−56.40	−15.40

**Table 4:** Representative Alignment Between Experimental Findings and Present Computational Trends.

Reference	Experiment Type	Key Finding	Alignment with Current Work
[28]	Optical/electronic characterization of doped CdS	Doped nanostructures show enhanced visible-light activity and reduced recombination via electronic structure tuning.	Matches rod-shaped CdS.
[29]	Review of CdS S-scheme for water splitting	Rods/nanowires balance crystallinity/surface area, reducing photocorrosion	Validates computational prediction of rod stability
[30]	Photocatalytic activity trends in pristine/doped CdS	Morphology dictates optical properties and activity; rods outperform clusters in stability.	Aligns with rod integrity under hydration (RDG/NCI)
[31]	Hydrothermal synthesis and RhB degradation	Nanorod-based hierarchical CdS shows superior activity from high surface area and crystallinity	Supports rod-shaped CdS balanced interactions

## Data Availability

All data supporting the findings of this computational study are reported in the manuscript.

## Supplementary Materials

The following supporting information can be downloaded at: https://www.mdpi.com/article/10.3390/molecules31010092/s1. The attached supporting material contains coordinate files for the rod- and cluster-shaped CdS structures used in DFT calculations, along with a Python version 3.10.12 script for plotting the radial distribution function from classical all-atom MD simulations.

## Abbreviations

The following abbreviations are used in this manuscript:

BCP	Bond critical points
CP	Critical points
DFT	Density functional theory
MEP	Molecular electrostatic potential maps
MD	Molecular dynamics
NCI	Noncovalent interactions
RDG	Reduced density gradient
RDF	Radial distribution function
