# Supplementary material for: Atomistic Insights into the Molecular Interactions of Rod and Cluster Shaped CdS for Photocatalytic Water Splitting
Source: Molecules. 2025 Dec 25;31(1):92. doi: 10.3390/molecules31010092 (PMC12786702; doi:10.3390/molecules31010092)
Supplement: Supplementary file 1 [file molecules-31-00092-s001.zip › molecules-4015283-supplementary.pdf]

Supporting material

Atomistic insights into the molecular interactions of rod and cluster shaped CdS for photocatalytic water splitting

Aliya Assilbekova[1], Irina Irgibaeva[1], Mirat Karibayev[2], Ayaulym Amankeldiyeva[2], Sergei Piskunov[3], Nurlan Almas[1], Galiya Baisalova[1,4,*], Anuar Aldongarov[1,*]

[1]L.N. Gumilyov Eurasian National University, 010000, Astana, Kazakhstan; asylaliya@yandex.kz (A.A.); irgsm@mail.ru (I.I.); enu-2010@yandex.kz (A.A.); nurlanalmasov@gmail.com (N.A.);
[2]Laboratory of Renewable Energy, National Laboratory Astana, Nazarbayev University, 010000, Astana, Kazakhstan; mirat.karibayev@nu.edu.kz (M.K.); ayaulym.amankeldiyeva@nu.edu.kz (A.A.);
[3]Institute of Solid State Physics, University of Latvia, 1000 Riga, Latvia; piskunov@lu.lv (S.P.);
[4]Astana Medical University, 010000, Astana, Kazakhstan; bajsalovagalia1@gmail.com (G.B.);

*Correspondence: mirat.karibayev@nu.edu.kz (M.K), enu-2010@yandex.kz (A.A.)

DFT optimized structure of rod shaped CdS:

Cd	-14.90324400	-1.96209300	3.29409700
Cd	-15.27845100	3.09511000	-2.35795900
Cd	-17.06147100	-3.99478000	0.19101000
Cd	-10.53144300	-1.85370300	2.52828800
Cd	-10.94301500	3.97254000	-1.70954400
Cd	-11.61963900	-5.08875600	-0.97618600
Cd	-6.14451700	-1.00204800	2.50427800
Cd	-6.69911400	4.91221700	-1.32198800
Cd	-7.68210200	-4.34408000	-0.89167900
Cd	-1.65792100	-0.75384500	2.46681300
Cd	-1.88619300	5.20482900	-1.21560000
Cd	-3.85421300	-3.31451400	-1.30477100

Cd	2.55999300	-1.15714100	2.20545600
Cd	2.96328200	5.24826700	-0.84664200
Cd	0.17356200	-3.13410000	-1.85537900
Cd	6.79726300	-2.14249400	1.86318900
Cd	7.70935700	4.15954800	-0.57225600
Cd	4.17428400	-3.37940500	-2.26996300
Cd	11.02634800	-3.15705100	1.75498600
Cd	11.06239600	5.14632500	0.69832600
Cd	8.18436000	-3.57354400	-2.60338400
Cd	15.10254000	-4.04925100	0.53895100
Cd	14.78975100	3.21324800	0.08206300
Cd	12.06424700	-4.35325700	-2.83111300
Cd	-12.97668300	1.86298700	1.80874400
Cd	-16.58583100	-0.43219700	-0.83738900
Cd	-9.03035000	1.96561100	1.84754300
Cd	-11.66744900	-0.23057700	-1.41686800
Cd	-4.42184300	2.52529200	1.75223000
Cd	-7.04894800	0.58375900	-1.55427800
Cd	0.03292400	2.35121400	1.66079400
Cd	-2.26840500	0.87321600	-1.71294400
Cd	4.59532200	1.63346600	1.59946700
Cd	2.26240700	1.02006400	-1.86727200
Cd	8.89572300	0.70593800	1.92304800
Cd	6.56344400	0.59637000	-2.00762300
Cd	13.36738800	-0.36573100	2.27647800
Cd	11.24411700	0.37295600	-1.46103000

Cd	17.08165800	-0.15570400	1.69199600
Cd	15.06964200	-0.96411700	-2.14070300
S	-15.16819900	0.11153500	1.49421200
S	-16.24820500	-2.73380600	-2.16877400
S	-10.97010300	0.33913700	1.08297900
S	-12.14483400	-2.76953800	-2.25497400
S	-6.60919600	1.15655000	1.03997300
S	-8.16393900	-1.85081600	-1.74341000
S	-2.27679400	1.24070800	0.86589900
S	-4.39740300	-0.73880700	-2.17064500
S	2.12103100	1.03575300	0.73160300
S	0.09072000	-0.47356300	-2.52299400
S	6.58411500	0.22503600	0.58453500
S	4.19992200	-0.65302600	-2.81943800
S	10.98873100	-0.57969300	0.94477700
S	8.75335200	-0.89249100	-2.62527400
S	15.07550700	-1.40073200	0.44100000
S	12.51641800	-1.61606700	-3.07543000
S	-13.30808900	3.64254700	-0.38634200
S	-14.14511500	0.60719000	-2.19230400
S	-8.96865700	4.25122100	0.17190700
S	-9.87956600	1.55197600	-2.41071100
S	-4.31096800	4.80029300	0.07208700
S	-6.09699700	2.76378100	-2.85465000
S	0.33698400	4.74681000	0.34589200
S	-2.13971700	3.25536500	-3.05446500

S	5.14422100	4.20622200	0.55337100
S	2.27890700	3.60061800	-2.83669300
S	9.03366900	3.44681100	1.78470000
S	6.51229800	3.15353000	-2.79086100
S	13.29145300	2.36706000	2.18242300
S	10.19389200	2.90419200	-1.31511300
S	17.29482400	2.45306800	0.62833500
S	13.90878100	1.52754400	-1.82294300
S	-17.12622300	-2.30695700	4.63726700
S	-12.47610400	-2.10964400	4.42840200
S	-14.63510500	-3.84423200	1.35582500
S	-17.64335900	2.00136700	-1.58852100
S	-15.27434400	4.93138300	-4.20171100
S	-18.51420900	-1.76689100	0.68671100
S	-18.37575300	-6.22433500	-0.16427000
S	-8.31781100	-1.52777700	4.05510800
S	-9.94070600	-4.06461800	1.01637400
S	-10.95891000	5.81132600	-3.56460700
S	-13.05391300	-7.22979500	-0.51768900
S	-3.88306800	-1.27647200	3.93815300
S	-5.73540700	-3.14941000	0.81047800
S	-7.44817100	7.31938900	-1.98763400
S	-9.39803000	-5.69584100	-2.45851600
S	0.37474000	-2.19272200	3.46765900
S	-1.68884900	-2.75204600	0.28293000
S	-1.94840700	7.78275600	-1.60341800

S	-5.39070100	-5.49937500	-1.84579600
S	4.46037000	-2.91973700	3.02235300
S	2.34993400	-2.79683300	-0.07995400
S	3.34542100	7.82328500	-0.86173400
S	-2.04588800	-3.67325600	-3.37004900
S	8.58872000	-4.00872500	2.65603000
S	6.35336400	-3.68093400	-0.50827700
S	9.12183000	6.39186300	-0.90815600
S	1.98663300	-4.88854400	-2.89472500
S	12.91909500	-5.09549000	1.76098400
S	10.29945800	-4.21521100	-0.75784000
S	11.41078500	6.48479900	2.94094400
S	5.99008200	-3.55565300	-4.30038900
S	17.34512500	-5.12428400	1.38461200
S	14.52166000	-4.99338000	-2.04235700
S	13.46299300	5.47760200	-0.59013000
S	9.85673500	-4.92639300	-4.32389600
S	-13.35701000	2.69112000	4.23686500
S	-9.06031100	2.65539800	4.38703200
S	-4.41189400	3.22950900	4.28495900
S	-0.56705900	1.66320400	4.17219900
S	3.74072100	0.80799500	3.99959900
S	7.76213400	-0.42426000	4.06701500
S	12.02801400	-1.97566700	4.07310100
S	15.65396000	0.03481700	3.99217900
S	19.43583800	-1.30346500	1.87355000

S	16.88531700	-1.73378100	-3.83729900
H	9.92448700	-4.11897700	-5.42448000
H	5.97421000	-4.87717200	-4.65535200
H	1.85295900	-4.87879100	-4.25485100
H	-1.99884400	-5.03546500	-3.48850600
H	-5.53281300	-5.53935700	-3.20431200
H	-9.13058900	-6.98179200	-2.07883200
H	-12.45975300	-8.07533300	-1.41720300
H	-17.35959900	-7.00709400	-0.64445500
H	15.25709500	-4.11619300	-2.79521800
H	10.07336300	-5.52870600	-0.45933500
H	6.37520800	-4.98828500	-0.11688200
H	2.38606200	-4.03647300	0.49857400
H	-1.51539600	-3.90574100	0.99590100
H	-5.31317900	-4.14473900	1.64545700
H	-9.87279500	-5.07766200	1.93010800
H	-14.57024900	-5.00875200	2.06749900
H	-18.17878200	-1.45210500	1.97557900
H	18.03215900	-3.98124500	1.70370300
H	13.36202000	-5.09016700	3.05494900
H	8.66458100	-3.73272300	3.99319700
H	4.68291800	-2.52061000	4.31113000
H	0.51576600	-1.62293900	4.70256300
H	-3.91850100	-0.16180100	4.72850700
H	-8.56327200	-0.25564000	4.50550800
H	-12.36462200	-0.89792400	5.05058100

H	-16.64985500	-2.18275000	5.91564100
H	15.89379200	-1.22059000	4.48425700
H	12.96799100	-2.82128600	4.59656900
H	8.80164300	-1.12128100	4.62107300
H	4.84448800	0.20496700	4.54069800
H	0.64172500	1.24242500	4.66149000
H	-3.15811800	2.78769700	4.62127900
H	-7.70878100	2.76499700	4.58742200
H	-12.05996400	2.90648500	4.62092600
H	-13.99852700	5.40785200	-4.05369400
H	-9.80886700	6.47066500	-3.21049500
H	-6.26758400	7.82967000	-2.45950000
H	-0.63339800	8.00716800	-1.91351900
H	4.68782200	7.84405500	-0.59159300
H	8.54640400	7.27884100	-0.04062800
H	12.14324700	7.54461000	2.47377300
H	14.04298300	6.39552700	0.24078300
H	13.83382300	2.22937800	-2.99030100
H	10.48866700	3.42446800	-2.54501500
H	7.57242900	3.14159200	-3.65398200
H	3.42803400	3.58826500	-3.57738700
H	-3.48467400	3.36094000	-3.30364200
H	-7.03227000	2.90875800	-3.84156800
H	-9.93751400	1.52277400	-3.77452500
H	-14.26248100	0.03379500	-3.42611700
H	-17.41476100	-2.79356500	-2.88201700

H	-13.50956500	-2.75449800	-2.36947500
H	-9.22650400	-1.82127900	-2.59703400
H	-4.43346700	-0.87025100	-3.52872700
H	0.01216100	-0.46198500	-3.88464900
H	4.19508900	-0.54817000	-4.17831000
H	8.96220200	-0.68878300	-3.95965500
H	12.42810200	-1.22947500	-4.38001700
H	17.76485400	-2.32157000	-2.96803400
H	-16.13141700	0.81236900	2.16532600
H	17.54971000	3.16448900	1.76759700
H	14.12015900	2.71758300	3.21248800
H	8.42859900	3.97971100	2.88554600
H	5.23062800	4.99631100	1.66290200
H	0.19619500	5.70357100	1.30901200
H	-4.40051400	5.84598000	0.94465900
H	-9.27637100	5.36486300	0.89712000
H	-13.52461400	4.87195800	0.16578100
H	-17.88602400	2.58228800	-0.37538100
H	19.44185900	-1.97809200	0.68165800

DFT optimized structure of cluster shaped CdS:

Cd	0.84036900	-3.36169600	6.11780200
Cd	-4.52799000	-5.91632500	4.62724300
Cd	4.15197700	-7.10787200	2.83296900
Cd	-2.96880400	4.03868600	4.68799100
Cd	-7.97285700	1.45130900	3.44733300

Cd	-2.76987000	8.40784400	1.06359300
Cd	5.37201000	3.10953400	3.10011500
Cd	8.99801700	-0.38224000	0.84070300
Cd	4.77959800	7.33980600	-1.32660900
Cd	0.27928700	0.34197000	1.26662600
Cd	-4.68242200	-2.34909000	-0.29198900
Cd	3.32293500	-3.27700700	-2.05569400
Cd	-1.41347700	-5.93349000	-4.47021800
Cd	-0.15252300	4.04258000	-3.21690200
Cd	-5.04497500	0.84056400	-5.57676700
Cd	3.11591600	-0.01735800	-6.67834600
Cd	-3.85496800	-2.74964100	-7.73922600
Cd	4.47757600	2.20394600	6.67272000
Cd	-1.15203500	0.29374300	5.36428700
Cd	-6.37880400	-2.36057600	3.83831600
Cd	7.27496900	-1.02700300	4.14368900
Cd	2.20130900	-3.46495000	2.04479000
Cd	-2.75333100	-6.01210800	-0.13564000
Cd	5.43767200	-7.01191500	-1.89009900
Cd	3.85638600	7.27459900	2.16883500
Cd	-1.51371900	4.16094900	0.64778500
Cd	-6.34632100	1.49362300	-1.17036500
Cd	-1.66845100	8.25522400	-3.37524200
Cd	6.74333200	3.41911900	-0.82248700
Cd	1.73189900	0.39862800	-2.65096200
Cd	-3.27558900	-2.76109400	-4.11177200

Cd	5.19672400	-3.79625700	-5.62134800
Cd	1.00522300	4.23083300	-7.14848700
Cd	-0.32592300	4.51626100	7.29758800
Cd	-5.68629700	0.76511800	6.13585300
Cd	2.80381300	-0.25951900	4.32943800
Cd	6.36597700	-3.74216600	0.98865800
Cd	-1.93972600	-2.84601200	2.76426800
Cd	-6.86654500	-5.47803900	1.33114000
Cd	1.51423200	-6.64747700	-0.63542400
Cd	-0.20351600	7.61581500	3.42122500
Cd	-5.57387700	4.87797400	1.44767500
Cd	2.64730800	3.59950300	-0.07559000
Cd	6.06097300	-0.34819600	-3.17301600
Cd	1.80705800	7.71304300	-4.36956100
Cd	-2.42171900	0.81281500	-1.95608200
Cd	-7.40690400	-2.08993000	-3.62645000
Cd	0.78358100	-2.58891300	-5.60848900
Cd	-2.67882500	4.08654800	-7.30102300
Cd	1.46173900	0.73906700	8.26191200
Cd	-3.77312200	-3.04532200	6.74708800
Cd	5.25664300	-4.47680300	5.05476500
Cd	-0.33833800	-6.54890500	3.53853300
Cd	1.15891900	3.54018200	3.77782700
Cd	0.91321100	7.64404300	-0.45091200
Cd	-3.78718300	0.94357200	2.13919400
Cd	-8.68063300	-1.51191400	0.71833700

Cd	-4.01713100	4.69987600	-2.96262300
Cd	4.54076100	-0.12921800	0.59289000
Cd	7.92246100	-4.37655800	-2.19831500
Cd	3.91118300	3.60781300	-4.15164600
Cd	-0.49114300	-2.86541400	-1.29030700
Cd	-6.35559200	-5.62261500	-3.14821300
Cd	2.54011600	-6.32264800	-5.25369300
Cd	-1.25331500	0.67971900	-5.93785400
S	2.15056500	3.39577800	7.76366300
S	-3.22590900	-0.32456900	6.79887000
S	4.85069500	-1.60179100	5.21643100
S	0.22010300	-4.05141500	3.62869100
S	-4.48554100	-6.65032900	1.70274900
S	3.79099000	-7.78721300	0.20221900
S	1.95704600	6.04857200	3.96438800
S	-3.50868100	3.55454200	2.21441600
S	-8.21879700	1.15144800	0.60049000
S	-4.00323600	7.17885800	-3.61211100
S	4.87618900	2.44447100	0.65435100
S	8.01656100	-1.56428800	-2.08966300
S	4.17065100	6.15523400	-3.87605200
S	-0.23922600	-0.30735000	-1.16097000
S	-5.29651200	-3.23804300	-2.62795500
S	2.86267100	-3.82075900	-4.51115000
S	-0.79490600	3.27278400	-5.58440100
S	3.29043000	2.26351800	4.41564900

S	-1.61664200	-0.27409000	2.88100000
S	-6.62307600	-2.93816100	1.25059700
S	-4.79430000	3.29103300	6.32765200
S	-1.28405400	-3.60100800	7.59736800
S	6.61050000	-1.23946900	1.58722300
S	1.55550400	-4.13432700	-0.37113400
S	-3.27418700	-6.56887900	-2.59906200
S	1.92763700	-7.69322700	4.06039400
S	2.97835300	6.07875100	0.04971300
S	-2.19833900	3.38946800	-1.74089300
S	-6.98571100	0.58776000	-3.54302500
S	-5.16600100	7.40258800	0.92715400
S	6.10454300	2.34510700	-3.30236800
S	1.13763400	-0.04908000	-5.07802200
S	-3.38614400	-4.67578700	-6.01667200
S	-0.74888000	2.85801700	5.37976000
S	-5.71415100	0.20534300	3.57343100
S	2.44071300	-0.90269200	1.91105600
S	6.61901500	1.77212300	5.08646000
S	5.41464900	-4.51207100	-1.34506000
S	3.10273200	-4.57807100	6.59523500
S	-2.43255900	-3.52209900	0.28153800
S	0.76801600	-7.14751500	-3.02598600
S	-2.44834300	-7.55448700	4.60010400
S	-0.95182400	6.57617100	1.13732600
S	-5.95977000	4.11428600	-1.14170200

S	2.10141500	2.93495000	-2.52746600
S	7.01112600	5.89930500	-1.40637600
S	5.39684100	-1.02724900	-5.68909900
S	-3.06791700	-0.16225500	-4.23553800
S	-1.40312600	-1.79214300	-7.20888800
S	1.07146000	-0.76247900	6.13793600
S	-3.95136600	-3.44071500	4.16158900
S	4.44876100	-4.57559700	2.54551300
S	-0.58449800	-7.20161800	0.91184700
S	0.62645100	2.88087300	1.36266500
S	5.84077100	5.62419100	2.89848600
S	-1.57334200	6.25569200	5.39414700
S	0.14487300	6.52032100	-2.76589000
S	-4.33340300	0.22935500	-0.31254200
S	-7.35223800	4.05231500	3.09997900
S	-4.78046200	3.45088700	-5.10549900
S	3.91618000	-0.75036200	-1.86271600
S	8.76607800	2.12000700	0.02893200
S	3.27335000	2.71379400	-6.61862800
S	-0.83902900	-3.52371200	-3.72588600
S	-3.36044700	1.51649800	-7.61521300
S	-7.09460900	-5.37846700	3.95345600
H	-8.10123200	-6.06356100	4.56022000
S	5.77804300	-7.19148500	4.83837800
H	7.03118700	-7.15298500	4.29299900
S	-8.24182200	1.61370200	6.07489100

H	-8.90335000	0.44527500	6.33246900
S	-1.54180500	9.46505000	-1.01164700
H	-1.61921600	10.82055700	-1.11204900
S	10.97686800	-1.68009100	-0.02049500
H	11.89984900	-1.45109300	0.96401900
S	5.51180500	8.83821600	0.77229300
H	4.75440900	9.95815000	0.56851000
S	0.20118500	-6.78387400	-6.33606700
H	0.00642900	-6.12700500	-7.51381800
S	-5.90096300	-1.56687000	-6.32230700
H	-6.81596900	-1.46970500	-7.33177400
S	2.13690500	-1.98694600	-8.00291900
H	3.12558900	-2.88520700	-8.28920100
S	-4.65108300	-2.61283500	-10.07678800
H	-3.69457400	-3.35729400	-10.71191100
S	4.08166300	0.71650700	8.72243500
H	4.68456200	-0.50206200	8.63445200
S	-9.08681100	-1.11823300	3.28076500
H	-10.31195400	-1.42651600	3.78857400
S	9.56814900	0.00100100	3.51276700
H	10.44675500	-1.00693100	3.79510100
S	7.94203300	-7.14711300	-2.48084300
H	8.57473700	-7.49899200	-1.32311100
S	1.69074100	8.95159200	1.96698100
H	1.85601200	10.30096500	2.03441300
S	-0.16395700	9.42961400	-5.14391500

H	0.12044300	10.64449600	-4.58941500
S	4.20487900	-5.51769900	-7.28440100
H	5.13217200	-6.43235200	-7.68836700
S	-0.63931500	4.09820100	-9.13426200
H	-0.57474000	5.28228700	-9.80878900
S	-0.82186100	5.75028200	9.37500700
H	-26.47789200	0.56860600	5.23965100
S	-6.32563900	-1.92452600	6.53636000
H	-7.40394700	-2.34040800	7.25536100
S	8.70236500	-4.68865300	0.31479900
H	9.61659200	-3.67165600	0.49673600
S	-7.48048300	-6.56369300	-0.90776000
H	-8.79611100	-6.29405400	-1.14710800
S	-2.12052000	9.47876200	3.35712500
H	-1.47319800	10.62478600	2.99326100
S	2.00547700	6.58827400	-6.77106800
H	1.18946100	7.35798800	-7.54981800
S	-8.50363900	-4.33419400	-4.30250200
H	-8.20729900	-4.52391600	-5.62208400
S	-3.95205200	6.24960300	-7.23598800
H	-3.26590200	7.08737900	-8.06964000
S	-0.63209000	0.57125300	9.60217400
H	-0.69549600	-0.79771600	9.64302600
S	-4.59603500	-5.54591500	7.26842600
H	-5.92987500	-5.39888900	7.52346400
S	7.66446200	-3.45300200	5.19755400

H	8.61990000	-4.22640200	4.60842600
S	2.77028400	8.99404800	-2.20124400
H	2.94611900	10.34382000	-2.20937900
S	-9.36639500	-1.72146200	-1.74518500
H	-10.08317100	-2.87424100	-1.88305500
S	7.62290000	-4.15924600	-4.85330700
H	7.91653200	-5.40992800	-5.31560400
S	-5.78790700	-7.23067700	-5.01056200
H	-6.98083700	-7.31729100	-5.67563200
S	4.15745800	-7.95175000	-3.93931900
H	5.20378500	-8.26972800	-4.75662300
H	2.43819700	3.83773900	9.02454900
H	-2.75515500	0.07216300	8.02548800
H	4.88160100	-1.35220800	6.55568400
H	-4.73950500	-7.98586600	1.59303200
H	3.58328400	-9.13088800	0.09509700
H	2.35559500	6.18238900	5.26244800
H	-9.37763100	1.65833800	0.09168600
H	-4.11067100	7.02433000	-4.98634000
H	9.14071300	-1.19782100	-2.77132700
H	5.08255600	6.57425500	-4.79969300
H	-5.90397000	3.95470100	5.88471100
H	-1.38857800	-4.90477000	7.98660100
H	-3.49638800	-7.91210200	-2.68287300
H	1.76509600	-9.04489100	4.08394200
H	-8.18451900	1.15302200	-3.86258000

H	-5.52671300	7.52909300	-0.38277200
H	7.14281400	2.73861700	-4.09439600
H	-4.49723800	-5.48985300	-5.84300300
H	7.84327100	2.26066800	5.43234500
H	2.78412600	-5.90458100	6.64774400
H	0.78125300	-8.50246700	-3.18295200
H	-2.12952300	-7.71205500	5.91791000
H	-7.13375200	4.67371500	-1.55009900
H	7.66719600	6.35808600	-0.29964100
H	6.45439400	-0.71244500	-6.48883900
H	-0.81622800	-1.71614500	-8.43939700
H	-0.83518000	-8.54121200	0.91658200
H	6.47602900	6.05599800	4.02343000
H	-2.35846900	7.19421700	5.99601600
H	-8.47733200	4.80994700	2.97352900
H	-5.81560600	4.16870400	-5.62676200
H	9.86316000	2.40554000	-0.72428800
H	4.23118000	3.21606600	-7.45086200
H	-3.37156100	0.92440700	-8.84068600

RDF analysis of rod-shaped and cluster-shaped CdS (Python script)

```
import numpy as np

from google.colab import files

uploaded = files.upload()

data = np.loadtxt("RDF")
```

```
# First column = r

r = data[:, 0]

plt.figure(figsize=(12, 4))


plt.plot(r, rdf_cd_o, label="Between Cd atoms of cluster and O atoms of water", linewidth=1.6)

plt.plot(r, rdf_s_o,  label="Between S atoms of cluster and O atoms of water", linewidth=1.6)


plt.xlabel("Distance, Å")

plt.ylabel("g(r)")

plt.title("")

plt.grid(alpha=0.3)

plt.legend()


# Set x-axis range from 1 to 6 Å

plt.xlim(1, 6)


plt.tight_layout()

plt.show()

import matplotlib.pyplot as plt


# Load RDF file

data = np.loadtxt("RDF", comments="#")


r = data[:, 0]
```

```
# Extract selected RDFs using 0-based indices:
# Cd–O → (1–3) → index 2
# S–O  → (3–4) → index 8

rdf_cd_o = data[:, 2]
rdf_s_o  = data[:, 8]
```
